# Functionalized MOFs for Subnanometric Control of Pd Speciation for Selective Hydrogenation of Butadiene

**DOI:** 10.1021/acsanm.6c00899

**Published:** 2026-06-15

**Authors:** Donato Decarolis, Sahra Ahmed, James King, Alin-Marin Elena, Linda Zhang, Jeff Armstrong, Ines Lezcano-Gonzalez, Mohsen Danaie, Michael Hirscher, Simone Meloni, Andrew M. Beale, Petra Á. Szilágyi

**Affiliations:** † 120796 Diamond Light Source, Didcot OX11 0DE, U.K.; ‡ Centre for Materials Science and Nanotechnology (SMN), Department of Chemistry, 6305 University of Oslo, P.O. Box 1033, N-0315 Oslo, Norway; § School of Engineering and Materials Science, 44617 Queen Mary University of London, Mile End Road, E1 4NS London, U.K.; ∥ Scientific Computing Department, 97005 Science and Technology Facilities Council, Daresbury Laboratory, Keckwick Lane, Daresbury WA4 4AD, United Kingdom; ⊥ Advanced Institute for Materials Research (WPI-AIMR), Tohoku University, Sendai 980-8577, Japan; # ISIS Pulsed Neutron and Muon Facility, Science and Technology Facilities Council, Rutherford Appleton Laboratory, Didcot OX11 0QX, U.K.; ∇ Department of Chemistry, University College London, 20 Gordon Street, London WC1H 0AJ, U.K.; ○ Research Complex at Harwell (RCaH), Harwell, Didcot, Oxfordshire OX11 0FA, U.K.; ◆ Diamond Light Source Ltd. Electron Physical Science Imaging Centre (ePSIC), Harwell Science & Innovation Campus, Didcot, Oxfordshire OX11 0DE, U.K.; ¶ Max Planck Institute for Intelligent Systems Solid State Research, D-70569 Stuttgart, Germany; †† Department of Chemical, Pharmaceutical and Agricultural Sciences, University of Ferrara, via Luigi Borsari 46, 44121 Ferrara, Italy

**Keywords:** metal−organic frameworks, heterogeneous catalysis, selective diene hydrogenation, hydride phase activity, nanoconfinement effect

## Abstract

Metal–organic frameworks have been intensively investigated for their ability to effectively control the growth and surface chemistry of nanosized guests, with their pores acting as templates and potentially providing anchoring sites. Since the speciation, as determined by the geometry and surface chemistry of hydride-forming metals, such as Pd, under particular conditions (*T*, *p*), is controlled by their size at and beyond the nanoscale, metal–organic frameworks are a prospective matrix for speciation or phase selection. This is of relevance because the role and characteristics of the phases in hydrogenation reactions involving hydride-forming Pd catalysts are open questions. In particular, it is a matter of debate which palladium phase is the most active and most selective, as they often occur simultaneously under catalytic conditions. For the first time, our thorough investigation, including *operando* XAFS and computer simulations, demonstrates that by embedding Pd nanoclusters, ≤1 nm in diameter, in the pores of the NH_2_–UiO-66 metal–organic framework, the speciation of subnanometric Pd particles can be controlled, such that the active particles only exist in their metallic state under reaction conditions; in fact, the Pd–H_2_ mixture only affords surface-bound hydrogen atoms. This control of Pd speciation consequently enables the direct probing of the phase activity and selectivity in the model reaction of 1,3-butadiene hydrogenation to butenes, wherein it showed no deactivation and improved selectivity compared to conventionally prepared catalytic systems. This result shows that the metallic phase can be stabilized through subnanometric size control and that it is more selective and less prone to overhydrogenating the butadiene reactant to butane, resulting in a purer product.

## Graphical abstract

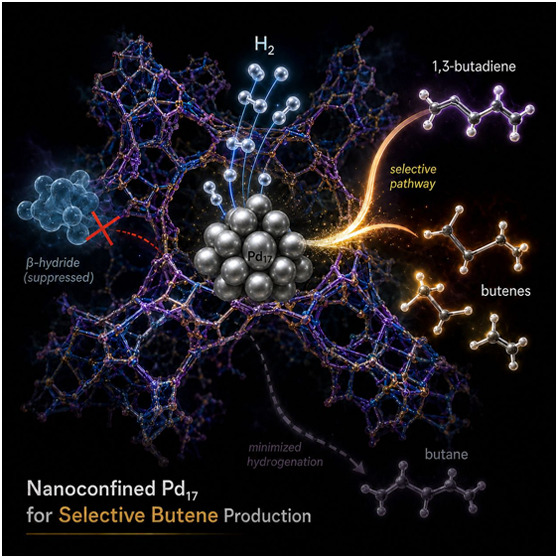

## Introduction

The design of highly active and selective heterogeneous catalysts has long been a major goal in catalysis science. For many hydrogenation reactions vital for the production of saturated hydrocarbon derivatives used as feedstock chemicals for numerous processes,^1–3^ palladium is an excellent catalyst. Palladium is able to reversibly split molecular hydrogen and subsequently absorb hydrogen atoms to form metal hydrides. At low concentrations, hydrogen atoms are exothermically absorbed in the Pd metal, forming a solid solution (α-phase) with largely unchanged physical properties of the metal.^4^ In this phase, the metal lattice undergoes expansion proportional to the hydrogen concentration, approximately 2–3 Å^3^ per H atom.^5^ Upon further increasing the hydrogen pressure at a given temperature, the so-called *plateau pressure* is reached, at which a phase transformation takes place. In the plateau-pressure region, the interstitial hydride, known as the β-phase of PdH_0.67_, is formed, and the gas pressure remains unchanged until the α-to-β transformation is completed. The interstitial hydride has noticeably different physical properties from the metal,^6–9^ including its optoelectronic properties; therefore, it has long been suspected that its chemical properties are also different. However, under catalytic conditions, the two phases typically coexist, making the assignment of different behaviors and performances to the individual phases challenging.^10^ Consequently, the role of these phases in hydrogenation reactions, both in terms of activity as well as selectivity, is still a matter of debate within the catalysis community.^11^ Furthermore, both the formation conditions and the very existence of the interstitial-hydride phase depend on the size of the palladium particles.^12–17^ Therefore, we stipulate that if a specimen that may only form one of the hydride phases (if at all) can be synthesized, the catalytic performance characteristics of the different phases may be unequivocally determined.

The formation of the β-phase is accompanied by a large volume expansion of *ca*. 10–20%, which builds stress within the lattice.^18^ When the particles are nanosized, their surface-energy term increases significantly; however, this surface-energy increase does not affect the two phases in the same way because of their different molar volumes, which means that the relative thermodynamic stabilities of the two phases shift significantly, such that with decreasing particle size, the formation of the more expanded interstitial-hydride phase becomes less favorable.^19,20^ In fact, such nanosize effects become increasingly pronounced with decreasing particle size. This increased size dependence occurs to the extent that in the nanocluster (NC) size regime (*i.e*., when the particle size is smaller than the de Broglie wavelength of an electron in the given material), only hydrogen absorption on the Pd particle surface and subsurface is preferred.^21^ As such, it follows that at a sufficiently small particle size, H can only occupy the surface and (possibly) subsurface positions, with no interstices available, which would be key to successfully attributing the catalyst performance to the distinct phases.

To this end, we have selected the model reaction of 1,3-butadiene hydrogenation. This reaction has strong industrial relevance, as clean butene streams are required for polymer production.^22^ Although palladium is currently regarded as the only commercially viable catalyst,^23^ conventional supported nanoparticulate Pd catalysts are prone to overhydrogenating butadiene into butane,^24–26^ an undesirable reaction from both an efficiency viewpoint, as well as because butane acts as a pollutant in the production of polymers.^27^ The reason for overhydrogenation is not trivial, particularly considering that the Pd phases present under reaction conditions are variable and ill-defined. According to recent research,^11,28–30^ this could be explained by the presence of interstitial β-palladium hydride, with evidence suggesting that it is more active but less selective than the α-phase. However, the reported experiment was inconclusive because of the coexistence of both phases.^11^ Considering that the α-phase is more common, it is not possible to guarantee that a system in which only the β-phase is present can be designed. Therefore, if the effect of phase selection is to be studied, one must design a Pd catalyst in which interstitial-hydride formation is inhibited, and thus only the metallic phase may occur.

Interstitial-hydride formation in Pd particles is highly dependent on their size,^21,31^ and to inhibit it, uniform Pd NCs of ≤1 nm in diameter are required to constrain the Pd particles to their metallic phase. The metal–organic framework (MOF) NH_2_–UiO-66 has been previously demonstrated to be able to incorporate highly dispersed Pd NCs in the required size range^32^; therefore, it is an ideal choice of test material. The UiO-66 structure comprises tetrahedral pores of 0.75 Å and octahedral pores of 1.2 Å,^33^ and the filling of the tetrahedral (small) pores consequently results in NCs. This MOF also takes advantage of the immobilization of the guest palladium NCs via charge transfer between the Pd atoms and N atoms of the amino functional group.^32^ We therefore selected this composite of Pd embedded in the NH_2_–UiO-66 framework as the ideal system to probe the active phase for the butadiene hydrogenation reaction. Furthermore, the NCs embedded in the MOF pores are less likely to form interstitial hydrides from a steric viewpoint, as this phase formation is accompanied by a large lattice expansion, which would be hindered by the surrounding pore walls of the crystalline framework.^19^ We therefore hypothesized that Pd embedded in the pores of NH_2_–UiO-66 cannot undergo β-hydride formation and, consequently, is a useful system to determine the role of Pd phases in selective 1,3-butadiene hydrogenation over Pd.

## Results and Discussion

In order to selectively probe the catalyst performance of metallic Pd, we embedded NCs in the pores of NH_2_–UiO-66 (Scheme 1), wherein they were strongly anchored through charge-transfer interactions between the Pd atoms and −NH_2_ groups, as previously revealed through XPS studies.^32^ Furthermore, according to previously reported transmission electron microscopy (TEM) imaging, the Pd NC dimensions are in line with the pore diameters.^32^ It is important to note that TEM is a local ‘number-averaged’ technique and as such it only yields information on a small sample area and thus it is not necessarily representative of the sample. In order to avoid any misinterpretation that may be the consequence of this, we also probed the particle size with extended X-ray absorption fine structure analysis (EXAFS), which yields information on the ‘volume’-averaged particle size. The EXAFS Fourier transform (Figure 1) shows a clear peak at 2.5 Å, which corresponds well to the classic Pd–Pd distance found in bulk palladium metal. When performing the EXAFS fit on the Pd-loaded NH_2_–UiO-66 under He atmosphere, the Pd–Pd coordination number (CN) was determined as 5.7, which, according to the method developed by Beale et al.,^34^ is consistent with a particle size of 7 Å, which is very close to the theoretical diameter of the MOF’s small pores (Table S1). Furthermore, the fit also suggests the presence of another coordination environment at a lower radial distance of 1.8 Å, which, when fitted with a Pd–N path, reveals a coordination number of 2. It is reasonable to assume that this Pd–N coordination is due to the interaction of Pd NCs with the NH_2_ functional groups present within the NH_2_–UiO-66 framework, in line with the previous XPS results.^35^ To verify such pore filling, we also embedded Pd NCs into the MOF pores computationally. First, the clusters were prepared according to the Wulff construction starting from an FCC Pd crystal, which provides the thermodynamically most stable (ideal) cluster of the given size. The geometry of the cluster was then optimized. The 7 Å particle diameter observed was found to correspond to the Pd_17_ NCs. It should be noted that this is in good agreement with our computational results, highlighting favorable filling of the small pores of NH_2_–UiO-66 with Pd_17_ nanoclusters, as well as a significant degree of immobilization through charge-transfer interactions between the Pd NCs and NH_2_ functional groups of the linkers. First-principles molecular dynamics simulations showed that (i) though very small, the Pd_17_ nanocluster is stable at the experimental temperature, and (ii) it strongly adheres to linkers in the small cavity. This binding is promoted by electron transfer (Figure SI1) from the Pd nanocluster to the linkers, resulting in an electrostatic attraction between the two. We will discuss this charge transfer in more detail in the following section when analyzing experimental results on the oxidation state of Pd. In summary, metallic Pd is primarily embedded in the tetrahedral pores of the MOF, *i.e*., they are of 7 Å in size and of the Pd_17_ formula; thus, they are true nanoclusters.

**Scheme 1:**
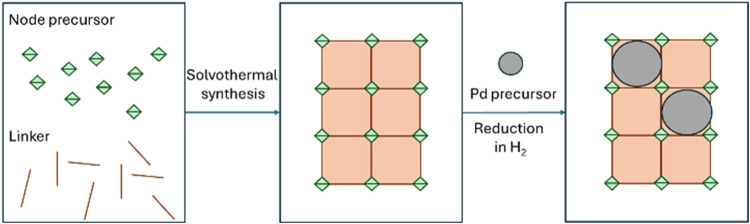
Schematic Representation of Sample Preparation

**Figure 1:**
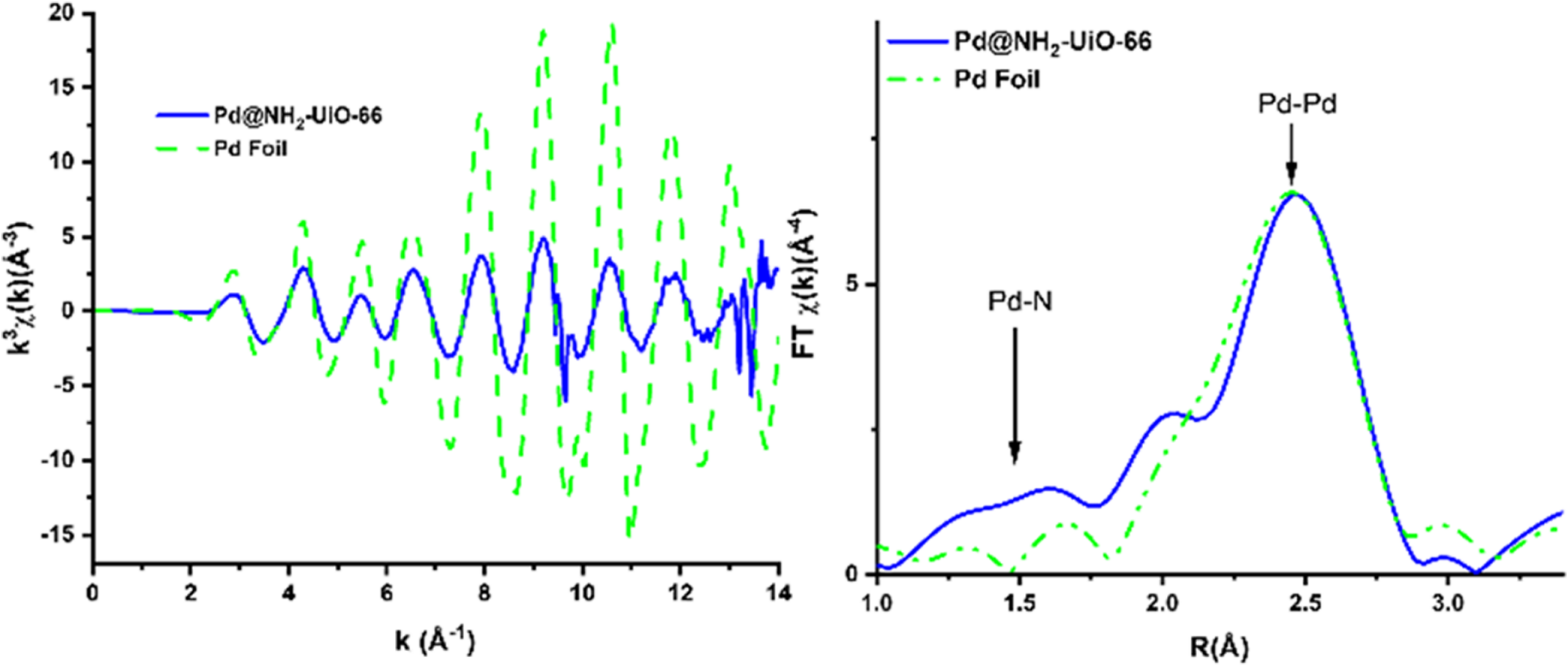
**Left**: *k* space. **Right**: *R*-space XAFS of Pd EXAFS of Pd-loaded NH_2_–UiO-66 against a Pd foil. Pd foil Fourier transform intensity was multiplied by 0.3 for comparison, with evidence of Pd–N and Pd–Pd contributions in the *R* space.

In order to prove our hypothesis on catalyst phase selectivity, we first set out to determine whether the hydride phase could form in these NCs using a combination of techniques. Thermal desorption spectroscopy (TDS) has been widely used to evaluate hydrogen desorption from metal hydrides and porous materials.^36–40^ Therefore, we measured hydrogen desorption from empty and Pd-loaded NH_2_–UiO-66 in the range of 20 K to room temperature, as shown in Figure 2. The samples were initially exposed to an H_2_–D_2_ mixture, cooled down to 20 K by using liquid helium, and then evacuated. By applying a constant heating rate, the hydrogen released from specific adsorption sites was determined using a mass spectrometer. Importantly, this method was shown to be able to differentiate between the metallic and hydride phases due to characteristic peaks of hydrogen desorption.^41^ For this reason, and as a means of comparison, TDS was also carried out on Pd NPs >1 nm (Figure 2a), in which the interstitial hydride was formed.

**Figure 2:**
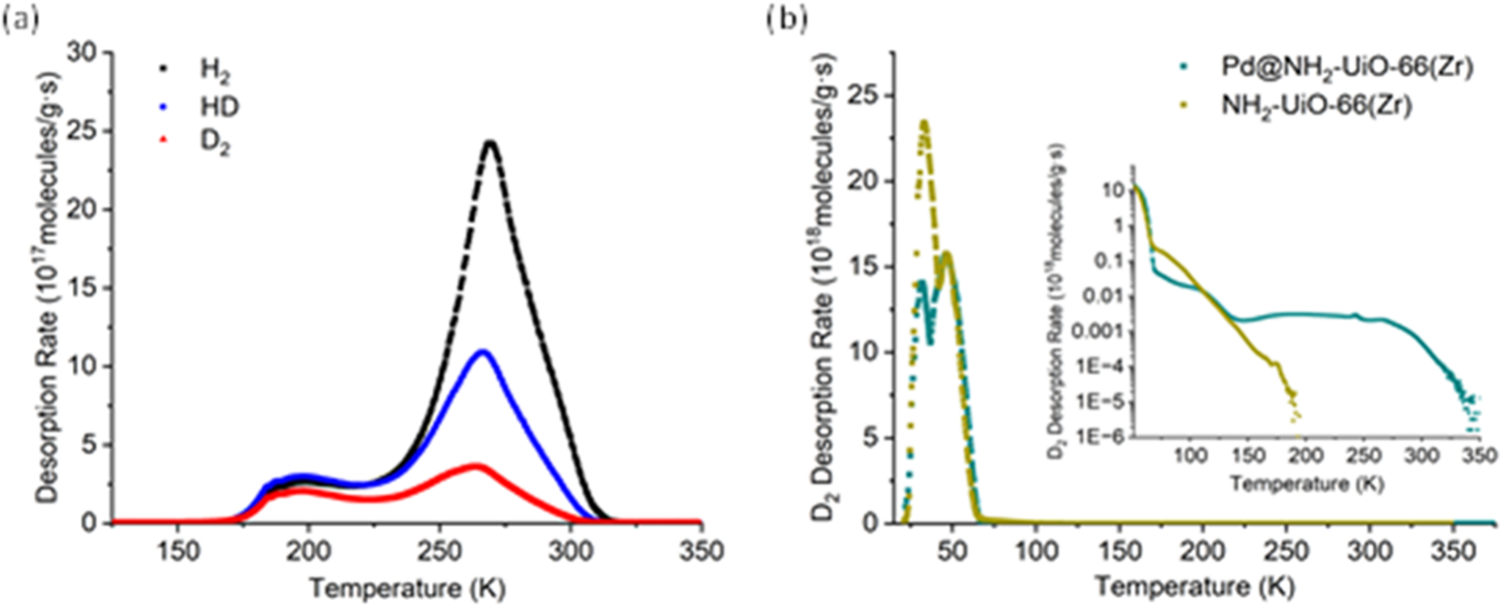
TDS spectra of 100 mbar 1:1 H_2_/D_2_ isotope mixture on (a) Pd NPs and (b) Pd-loaded and empty NH_2_–UiO-66 measured at 20 K with a heating rate of 0.1 K s^–1^.

The hydrogen desorption signal from Pd NPs (Figure 2a) shows the characteristic features of a sharper, higher-intensity signal superimposed on a broader, lower-intensity one, corresponding to H_2_ desorption from the different hydride phases (interstitial hydride and solid solution). This can be rationalized well in terms of the better-defined nature of the interstitial hydride in terms of its composition and structure. Our data consisted of a relatively intense signal centered at *ca*. 260 K, overlapping with a broad signal with an onset temperature of *ca*. 170 K, is well in line with that observed previously,^42^ with some expected particularities of the desorption temperature (a function of both particle size and ramping rate), as well as the relative areas under the desorption signal, which is related to the particle size due to the different hydrogen-absorption capacities of the solid-solution and interstitial-hydride phases. In order to evaluate the desorption data from Pd embedded in the pores of an MOF, we first need to extract the desorption data corresponding to Pd NCs. We followed the methodology established previously,^43^ in which data sets obtained for the Pd-loaded and empty MOFs were compared. Indeed, from this comparison, we observed that (Figure 2b, inset) there is desorption from the Pd-loaded sample through to 300 K, while there is no desorption from the empty framework above 200 K. This is because hydrogen is physisorbed on the MOF, while it is chemisorbed on Pd, so its energy of adsorption, and consequently, its desorption temperature will be distinguishable, and indeed in very different regions of the experimental data sets. This allows us to reliably analyze hydrogen desorption from only the Pd particles. When comparing the desorption data from the Pd NPs with that from the Pd-loaded NH_2_–UiO-66, it was apparent that the characteristic features observed for the NPs were absent. Instead, a very broad and low-intensity desorption signal with an onset temperature of *ca*. 130 K was observed. This broad signal is associated with a lower and less well-defined hydrogen loading, as would be expected from hydrogen being desorbed only from the metallic phase.^13,15^ We would like to point out that this observation was highly reproducible (Figure SI2), regardless of the initial hydrogen loading conditions (*T*, *p*), and thus we assigned it to an intrinsic property of the Pd NCs embedded in the MOF pores. We note that, despite no signal indicative of desorption off the interstitial β-hydride being observed (as was the case for the larger Pd NPs, for example), there was still desorption from the Pd independent of that from the MOF, which would normally be associated with hydrogen desorption from the α-phase, including hydrogen from bulk, subsurface, and surface binding sites.

Concerning hydrogenation reactions, we expect that hydrogen atoms in these different sites would react differently; specifically, the particular role of subsurface hydrogen has been suggested previously.^44^ In fact, the small Pd_17_ NCs of 7 Å, as observed previously with TEM and herein with EXAFS, are not expected to afford a significant number of absorption sites in their bulk owing to their small size and lack of interstitial sites; rather, we expect that absorption could predominantly occur on the NC surface and subsurface. The possible inner and surface binding sites for hydrogen are shown in Figure SI3. In order to verify whether both these absorption sites (*i.e*., surface and subsurface) are occupied by hydrogen (and thus whether a solid solution is indeed formed), we combined density functional theory (DFT)^45^ calculations with inelastic neutron scattering (INS).

INS spectra of both the empty and Pd-loaded MOFs *in vacuo* were acquired under H_2_ pressure at base temperature and are displayed in Figure 3. The Pd-loaded MOF sample was produced in a much smaller quantity than the pure MOF sample; thus, the signal strength from INS was much smaller. This much weaker signal made it much more challenging to distinguish smaller peaks from the background for the Pd-loaded MOF. Nonetheless, it is clear that the presence of Pd within the MOF significantly perturbs the phonon spectra, with the majority of modes either disappearing, shifting, or changing in amplitude. These major changes are likely due to the strong binding of the linkers to Pd as well as the effects of steric hindrance on the motions. The two most prominent modes occur at a low energy. The first occurs at ∼117 cm^–1^ and is attributed to the rotational mode of H_2_.^46^ This line occurs at ∼118 cm^–1^ in solid H_2_ and is the mode for the free rotation of molecular hydrogen. The same peak appears in general when molecular hydrogen is bound to a surface/interaction site; however, due to the electron loss/gain and the associated shortening or lengthening of the H_2_ bond, the position of this peak may be shifted up or down in energy. This is due to molecular hydrogen bound to the MOF adsorption site. The peak was also present for the H_2_-dosed MOF, with a smaller amplitude relative to the predosed sample when compared to that of the Pd-loaded MOF. One possibility is, therefore, that molecular hydrogen is bound to both the framework and Pd nanoparticles in the latter case. Also interestingly, we observed a large peak at *ca*. 240 cm^–1^, which was not present in the spectrum of the interstitial-hydride PdH_0.67_ (Figure SI4), observed on commercially available Pd nanoparticles, or indeed in the spectrum of the empty MOF under the same pressure of hydrogen atmosphere (Figure 3 Left). The mode, being too low for a simple H hydride displacement, is much more likely to be that of an indirect motion of H or H_2_ as a collective one involving H and/or Pd@MOF contributions. DFT phonon calculations were performed to aid in the spectral assignment and are discussed below. Also, it should be noted that the interstitial β-hydride spectrum, when compared with that of the pure metal, only displays the emergence of the well-known peak at *ca*. 465 cm^–1^ assigned to Pd–H stretching in the interstitial hydride.^45^ We could not detect this peak in the Pd-loaded MOF; however, care must be taken as the data quality may not allow for its detection in this case. For this reason, it cannot be ruled out solely on the basis of INS investigations, and our results must be discussed in light of the previous TDS results and additional experimental and theoretical investigations.

**Figure 3:**
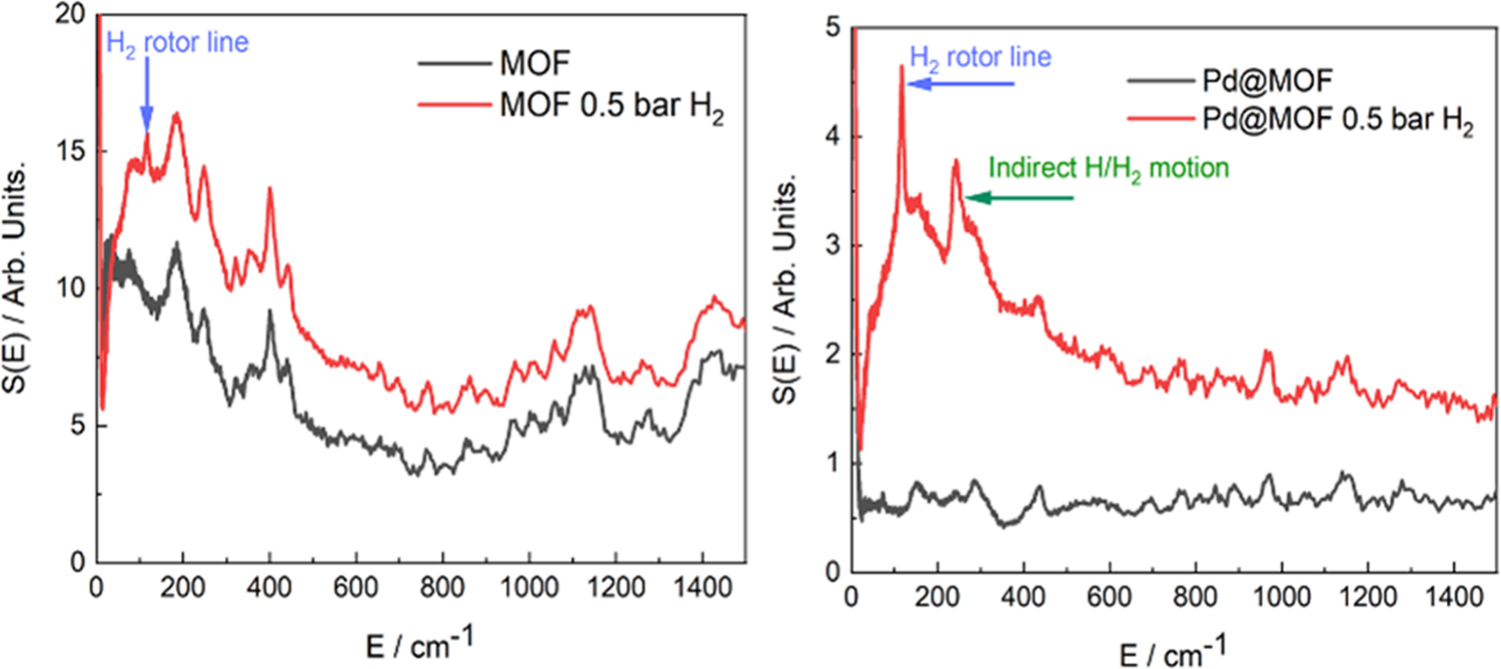
**Left**: INS spectra of pristine MOF, both empty (black) and with 0.5 bar of dosed H_2_ (red). The spectra are mostly similar; however, the H_2_-dosed sample shows a sharp peak at ∼117 cm^–1^, which is the characteristic value of the solid rotor of H_2_. **Right**: INS spectra of Pd@MOF, both empty (black) and with 0.5 mbar of dosed H_2_ (red). The H_2_-dosed sample shows a peak that was attributed to indirect H/H_2_ motions at ∼240 cm^–1^.

In order to evaluate the hydrogenation ability of the Pd-loaded MOF sample, we began by probing the hydrogen absorption of the Pd_17_ clusters computationally (Figures 4 and SI5). This cluster size was selected, as it corresponds to the 7 Å particle diameter observed experimentally. The clusters were constructed in the same way as described earlier. We identified two possible surface sites: Pd_△_ and Pd_◇_, referred to as hollow and FCC, respectively, and one subsurface site for hydrogen chemisorption. Our DFT calculations show that atomic hydrogen is preferentially accumulated on the surface of the Pd-loaded cluster in the FCC and hollow positions (Figure SI3); *i.e.*, it is exclusively absorbed on the cluster surface and not on the subsurface sites. More importantly, we have specifically probed whether the hydrogen absorption can be energetically favorable in the latter subsurface sites by computationally depositing H atoms into these positions. We found that the hydrogen atoms spontaneously moved toward the surface; in conclusion, subsurface sites are unstable absorption sites (see Supp. Info Video S1). These simulations were performed for Pd_17_ NCs embedded in the NH_2_–UiO-66 pores. Although our results may contradict previous assumptions on the viability of subsurface sites in the relevant size regime,^20^ the different behaviors may be due to matrix effects of the host framework. Nevertheless, we would like to point out that such small Pd NCs are unstable unless they strongly interact with a support because of their large surface energy; *i.e*., it is virtually impossible to experimentally probe hydrogen absorption on the subsurface sites of naked Pd_17_ NCs (even if they could be synthesized without a strongly binding support). In summary, our computational results support our experimental data, which suggest that the β-phase is unstable in the Pd NCs embedded in the pores of NH_2_–UiO-66. Furthermore, they suggest that the subsurface absorption sites are also unstable in this sample. Considering the changes observed in the INS spectrum on hydrogen absorption in the Pd-loaded framework, it is interesting to determine the mode corresponding to this band. In an attempt to identify the mode at *ca*. 240 cm^–1^, we performed further atomistic modeling to simulate the INS spectra *via* phonon analysis (Figure SI6). By comparing the computed phonon spectra of Pd-loaded NH_2_–UiO-66 before and after the absorption of one H atom per Pd NC, we observed that the presence of surface-absorbed hydrogen enhanced the intensity of the phonon spectra at low frequencies (<250 cm^–1^), which, although in good agreement in energy, did not share the same strong amplitude that we observed experimentally. These low-frequency modes appear to show H movement coupling with Pd, resulting in an increase in the spectrum intensity, consistent with the large displacements and low mass of H. This mode may be assigned to a collective mode involving surface-absorbed H, the Pd NC, as well as both the inorganic node and functional group of the host metal–organic framework (Video S2 in SI). Although this may form a portion of the observed peak at *ca*. 240 cm^–1^, the majority of the signal may be coming from some other form of collective motion, probably involving a large number of H/H_2_ moving collectively, which was beyond the scope of our DFT study.

**Figure 4:**
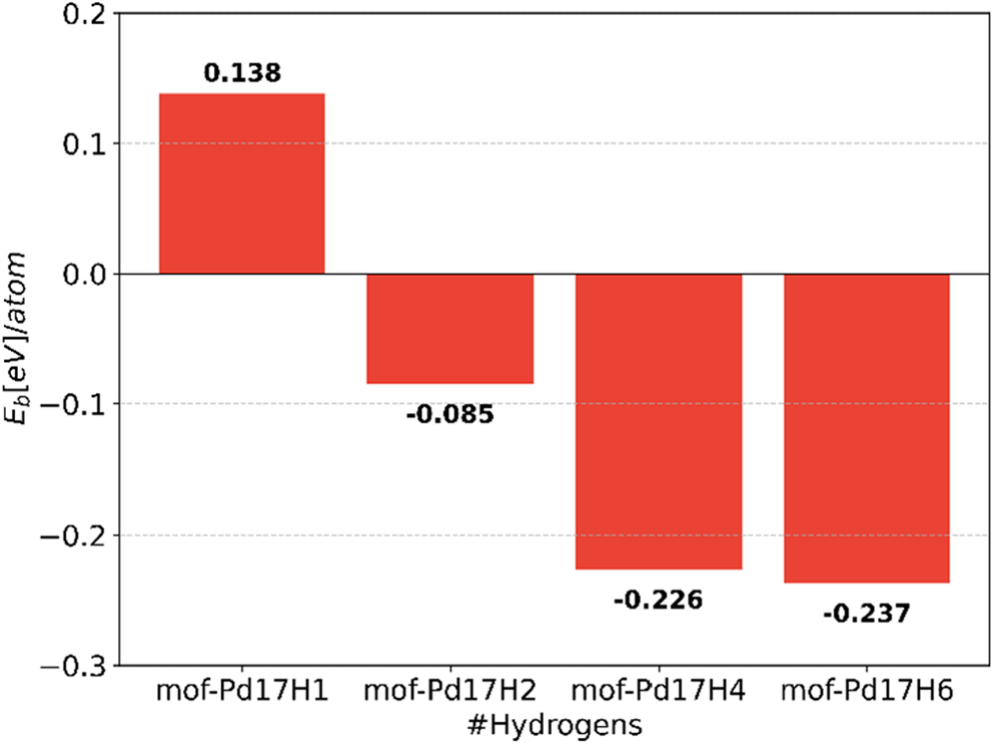
Binding energy per H atom clearly shows that the binding energy is more or less the same for all configurations with H on the surface when the cluster is embedded in the MOF.

To gain insight into the hydride formation on Pd_17_ nanoclusters embedded in NH_2_–UiO-66, we analyzed the energy of hydrogen absorption using DFT.

As mentioned before, the formation of β-hydride is accompanied by substantial volume expansion because the H atoms occupy the interstices. Nevertheless, it is reasonable to assume that even surface-bound H atoms may affect the cluster volume, particularly given their small dimensions. In order to assess the volumetric expansion of the 7 Å-sized Pd nanoclusters embedded in the pores of NH_2_–UiO-66 during hydrogenation, we carried out further X-ray absorption analysis.

By exposing the Pd-loaded MOF to a hydrogen pressure of 1 bar at room temperature, a shift in the multiple scattering peaks in the X-ray absorption near-edge structure (XANES) at 24,390 and 24,420 eV was observed Figures 5 and SI7. Such a shift is compatible with the degree of volumetric expansion of the Pd nanoclusters. Subsequent fitting of the EXAFS data provides information on the Pd–Pd distance within the nanoclusters, which corresponds to an increase from 2.74 Å in the as-prepared NCs to 2.78 Å upon exposure to H_2_ (Table SI1), *i.e*., to a *ca*. 1.4% radial expansion. Indeed, our DFT modeling also results in a volumetric expansion when H atoms are chemisorbed on the Pd nanocluster surface (Figure SI8). Such a (1.4%) expansion of the Pd–Pd nearest-neighbor bond lengths would correspond to the formation of Pd_17_H_6_ species (PdH_0.35_), as shown in Figure SI9. We underscore, however, that this is a rough estimate and probably an overestimate, and it does not consider slightly larger particles embedded in the octahedral MOF pores or potentially stranded on the MOF surface.

**Figure 5:**
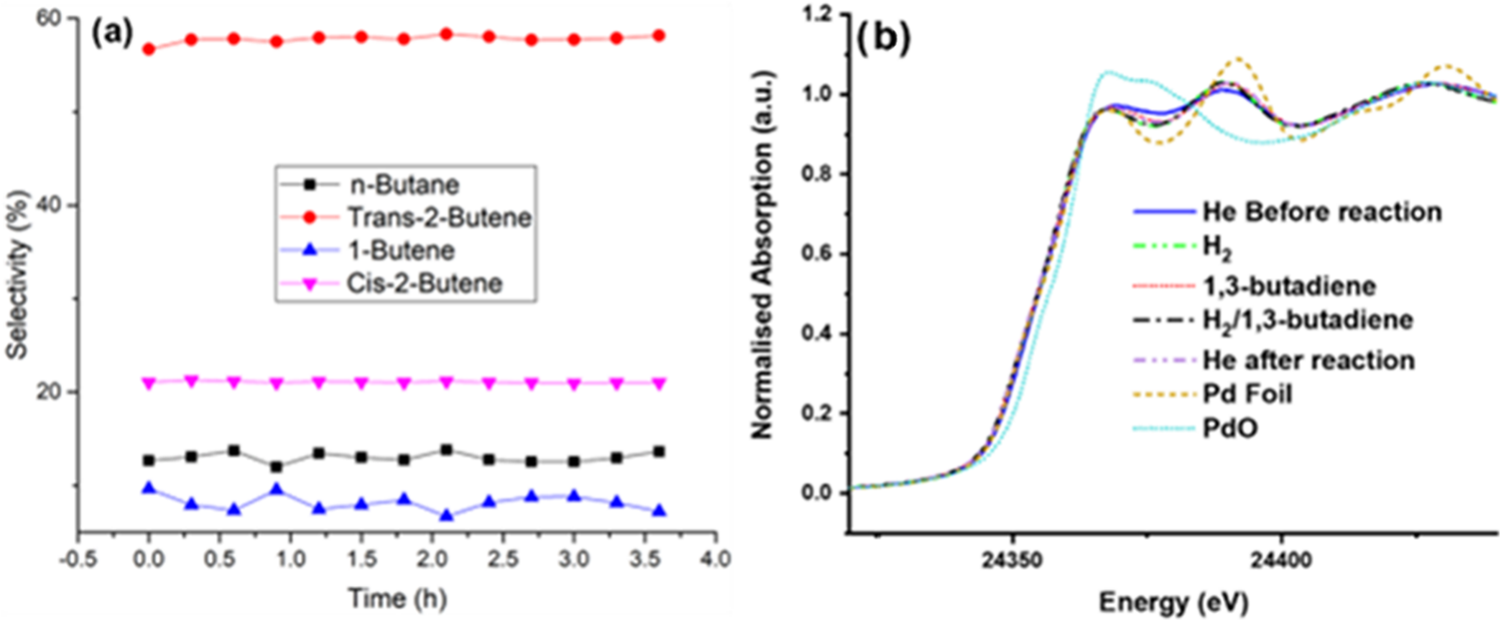
(a) Evolution of the selectivity for 1,3-butadiene hydrogenation over Pd-loaded NH_2_–UiO-66 as a function of time on stream, and (b) operando XANES spectra at the Pd K-edge for the reaction at room temperature together with PdO and Pd^0^ references. The reaction was performed at 298 K, the H_2_ to 1,3-butadiene ratio was 95.2:4.8, and the GC sampling time was 0.3 h.

It is therefore clear that such volume expansion can arise only from the hydrogenation of surface sites without the occurrence of a metallic to interstitial phase transformation. In fact, it does not even necessitate the absorption of H atoms on subsurface sites, which the Pd_17_ in the NH_2_UiO-66 sample cannot afford. This indicates that the Pd-loaded NH_2_–UiO-66 is the ideal sample to isolate the catalytic performance of the metallic phase of Pd, without any potential interference from hydride formation. Therefore, we were confident that we could proceed with the catalytic performance testing.

The catalytic activity of Pd-loaded NH_2_–UiO-66 for the hydrogenation of butadiene was investigate to probe the selectivity of the metallic phase. The reaction was carried out at room temperature, and the exhaust stream was monitored using gas chromatography (GC) (Figure 5a).

The catalytic activity and selectivity of the Pd NCs embedded in the pores of NH_2_–UiO-66 are remarkable. It converts 1,3-butadiene completely (100% conversion), with a combined conversion to butenes of 88% and minimal overhydrogenation to *n*-butane of ∼12%. This is quite noteworthy as other known Pd-based catalytic systems typically show a tendency to overhydrogenate with a high selectivity for the undesirable *n*-butane.^48^ This upholds the hypothesis that the hydride phase is less selective and more likely to result in overhydrogenation and demonstrates that a catalyst where only the more selective metallic phase is present shows superior catalytic performance. Within this context, we must note that on Pd-based catalysts, secondary metals need to be added to improve butene selectivity.^47,49^

In addition to the high selectivity, the catalyst also shows different behaviors compared to other studies on Pd-supported nanoparticles.^11^ In those systems, the catalyst displays high activity, paired with low selectivity, at the start of the reaction, which gradually decreases over time, in conjunction with an increase in butene selectivity. However, the Pd-loaded NH_2_–UiO-66 shows no sign of deactivation even after 4 h on stream.^11^ Previous literature data suggested that the cause of deactivation was the slow depletion of the interstitial-hydride phase, which is responsible for the overhydrogenation of butadiene to butane. In the Pd-loaded NH_2_–UiO-66, the absence of an easily depletable β-phase does not allow for this behavior to occur, thus preventing both the deactivation and overhydrogenation. We also assume that any potential deactivation by particle sintering was also prevented by the stabilizing effect of the MOF matrix on the Pd NCs.

We also collected *operando* X-ray absorption fine structure (XAFS) data (Figure 6) at the Pd *K*-edge to further observe the interaction of the system with reactive gases. Comparing the catalyst to a Pd foil, it can be seen that the white line, at 24,367 eV, is higher compared to that observed in the Pd metal, while it also displays a lower intensity of multiple scattering peaks at 24,390 and 24,430 eV (Figure 5b). This suggests the presence of nanoobjects smaller than 3 nm, as expected.^50,51^

**Figure 6:**
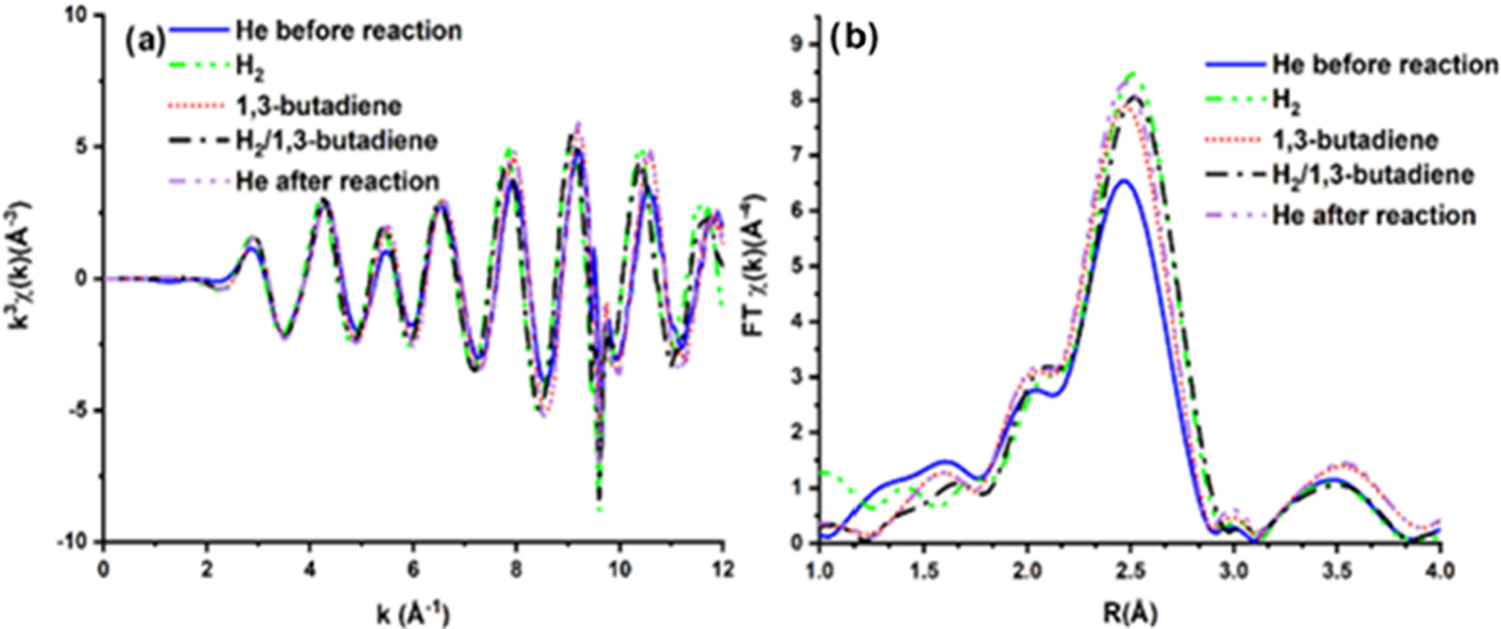
(a) *k* space. (b) *R* space XAFS of Pd, as a function of gas composition during 1,3-butadiene hydrogenation at room temperature: He (before reaction); hydrogen; 1,3-butadiene; gas mixture of H_2_/1,3-butadiene; He (after reaction).

Upon the introduction of hydrogen, an increase in the particle size may be observed, concurrent with the disappearance of the previously observed Pd–N contribution (Figure 6). When 1,3-butadiene is introduced into the system, the Pd–Pd distance reverts to that of a pure metal phase (2.74 Å), indicating that the surface-bound hydrogen atoms readily react with 1,3-butadiene and are thus consumed. However, under reaction conditions (H_2_/1,3-butadiene = 95.2%/4.8%), this behavior is not observed, as the surface sites are instantly replenished, confirming that it is the active phase for the hydrogenation of 1,3-butadiene and that the active surface-bound H atoms may be derived directly from the gas phase and do not need to be replenished from the subsurface sites.

It is noteworthy that, in addition to inhibiting the formation of the interstitial hydride, Pd-loaded NH_2_–UiO-66 also differs from other catalytic systems in terms of its well-defined charge-transfer interactions between the Pd NCs and amino groups, as evidenced by the XANES of the pristine catalyst, (Figure 5b), showing a higher “white line” than the sample exposed to H_2_ and our previously reported XPS data.^32^ To validate the hypothesis of charge transfer between Pd NCs and linkers, we analyzed the electronic density of the sample in which a Pd_17_ nanocluster was embedded in the MOF (Figure SI1). Bader charge analysis^52^ allows one to count the number of electrons belonging to each atom and, hence, the overall electronic charge belonging to either subsystem. This revealed that upon the embedment of Pd_17_ in NH_2_–UiO-66, almost one electron was transferred from the nanocluster to the MOF. This is consistent with the XPS results as well as the observed 1.8 Å Pd–N bond length reported above.

In addition, to assess the effect of the host–guest interaction on the catalytic performance, an additional experiment was performed to verify the stability of the Pd–N bond toward other parameters, such as temperature (Figure SI9). A fresh sample was heated to 200 °C while flowing He, and an increase in CN was observed, similar to that previously observed after the system was flushed with H_2_ at room temperature. This suggests that the Pd–N bond is fairly labile and can be broken by either temperature or reactant gases. Interestingly, the Pd–Pd bond length increases slightly from 2.74 to 2.77 Å after being exposed to such high temperatures, and this interatomic distance then remains stable. This has not been previously observed when the system was under reaction conditions at room temperature, which suggests a degree of reshaping of the nanoclusters compared to that observed previously upon the introduction of hydrogen. For both the thermally and chemically treated systems, we observe the disappearance of the Pd–N contribution, simultaneously with an increase in the Pd–Pd coordination number, suggesting the dissociation of Pd from the NH_2_ moieties. This was further confirmed by atomistic simulations, which also showed an increase of the Pd–N interatomic distance by 0.03 Å, *i.e*., bond weakening or even breaking upon hydrogenation. This behavior can be explained by the detachment of the nanoclusters from the functional groups in the NH_2_–UiO-66 framework, likely caused by the scission of the Pd–N bond. This hypothesis was confirmed by our TEM investigation, which demonstrated that the Pd particles retained their <1 nm size (Figure 7) upon thermal aging, corresponding to the nanoconfinement of Pd_17_ nanoclusters within the MOF pores. This suggests that the confinement of the nanoclusters within the framework is effective, thus confirming the high stability of the system toward external perturbations. However, there is evidence of the existence of some larger Pd nanoparticles in the sample, but neither their size nor their concentration is seemingly affected by the thermal process (Figure SI10). However, it should also be noted that, as evidenced by the lack of a diffraction peak corresponding to bulk Pd (Figure SI11), the concentration of these larger particles must be negligible.

**Figure 7:**
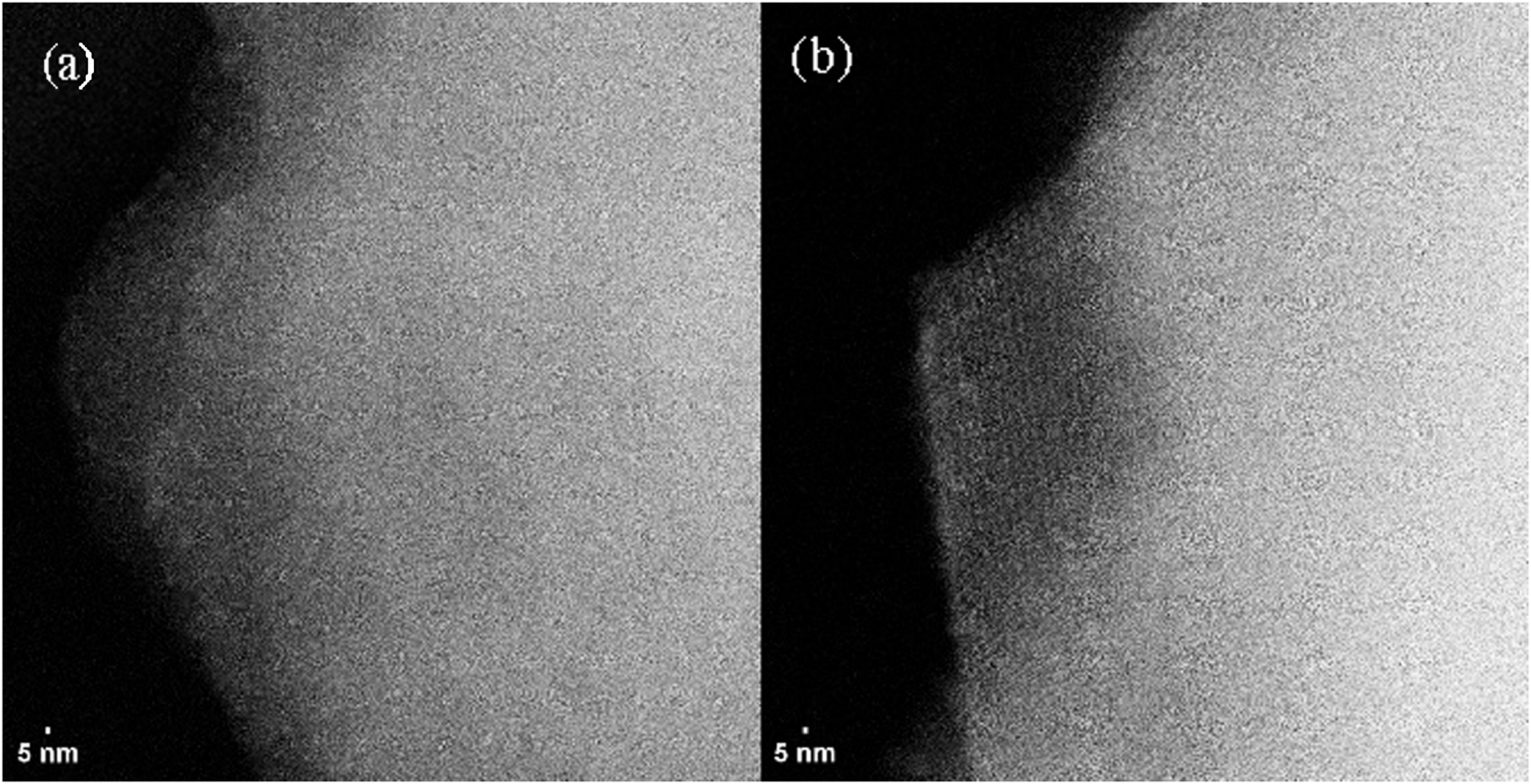
HAADF-STEM micrographs of (a) postreduction and (b) “aged” Pd-loaded NH_2_–UiO-66, *i.e*., heat treatment at 240 °C in He, showing Pd nanoparticles of <1 nm.

This insight explains that the only reason for the superior performance behavior is the lack of the β-phase in Pd-loaded NH_2_–UiO-66, as shown by our combined theoretical and experimental evaluation, rather than a consequence of a strong binding support effect. Remarkably, upon flushing the system with He after the reaction, the NCs return to the pure metal form, which suggests that the hydrogenation of the “hollow surface sites” is only stable under reaction conditions, *e.g*., when the sample is exposed to hydrogen.

## Conclusions

In conclusion, we have devised and designed a model system of NH_2_–UiO-66 loaded with Pd nanoclusters, in which we can isolate and determine the catalytic performance of the purely metallic phase of Pd in the selective hydrogenation of butadiene. With a combined experimental and theoretical approach, we show that in subnanometric Pd particles of 7 Å in diameter, no formation of the interstitial-hydride β-phase occurs, and even subsurface sites are not favorable to be occupied by the absorbed H atoms. Pd-loaded NH_2_–UiO-66 has been shown to be an active and stable catalyst for the 1,3-butadiene hydrogenation reaction, and importantly, it shows a selectivity of 88% toward butenes at room temperature when compared to conventional supported nanoparticulate Pd catalysts tested under the same conditions,^11^ thus proving our hypothesis. This demonstrates that the absence of the interstitial-hydride phase increases the selectivity of palladium for the hydrogenation of butadiene to butenes when compared to other Pd systems under the same reaction conditions.^54^ Furthermore, our data suggests that the minute engineering of nanocluster morphology by embedding into MOF pores not only hinders hydrogen absorption in the interstitial sites but also makes the subsurface sites inaccessible under reaction conditions. This is an important insight into understanding hydrogenation reactions, as the previously hypothesized subsurface absorption sites are crucial for catalyst activity.

## Materials and Methods

### Syntheses

NH_2_–UiO-66 was synthesized solvothermally, according to the process described by Farha et al. A 25 mL reaction vial was loaded with ZrCl_4_ (0.75 mmol, 174.8 mg), followed by 5 mL of dimethylformamide (DMF) and 1 mL of concentrated HCl. 2-Amino-benzenedicarboxylic acid (0.54 mmol, 97.8 mg) was dissolved in a separate vial containing 10 mL of DMF. Both vials were sonicated before being combined and heated at 80 °C overnight. The product precipitated out of solution and was filtered by centrifugation, washed twice with DMF, and then twice with EtOH. The samples were activated *in vacuo* at 125 °C overnight.

Pd was embedded in the MOF pores, as reported by Szilágyi et al.^32^ by first infiltrating an anhydrous acetonitrile solution of Pd­(NO_3_)_2_, 10 mg in 7 mL, into the pores of 100 mg of activated MOF, followed by its reduction in a 5 vol% H_2_/Ar mix at 150 °C for 1 h at 1.2 bar. The embedding resulted in a surface area and pore volume reduction of *ca*. 15% (Figure SI12 and Table SI2). The Pd content was quantified using microwave plasma atomic-emission spectroscopy and was found to be *ca*. 3 wt % Pd (Tables SI3–S6).

Pd nanoparticles (>25 nm) were purchased from Sigma-Aldrich (no. 686468) and used as a reference for TDS and INS spectra acquisition.

### Thermal Desorption Spectroscopy

Thermal desorption spectra of the 1:1 H_2_–D_2_ mixture were acquired using the setup described by Panella et al.^52^ 2.4 mg of activated samples (470 K, 3 h, <10^–5^ mbar) were exposed to 50 mbar of the 1:1 H_2_–D_2_ mixture at 298 K for 30 min and then slowly cooled down to 20 K. The temperature was maintained for 5 min, and the reactor was evacuated. A temperature program of 20–400 at 0.1 K s^–1^ was finally run. This control allows the circumvention of kinetic effects by setting the heating rate low, *i.e*., near equilibrium conditions. The high sensitivity of the quadrupole mass spectrometer allows the detection of desorbed hydrogen over several orders of magnitude.

### Inelastic Neutron Scattering

INS measurements were performed using a TOSCA indirect neutron spectrometer at the ISIS Neutron and Muon Source, Harwell, UK. Samples were loaded into Al gas handling cells within a glovebox before being attached to a gas manifold, where the cell was evacuated. Samples were first measured at base temperature (∼14 K) while empty before being reheated and dosed with H_2_ at room temperature before being cooled back down to base temperature and remeasured. Measurements at low temperatures ensured the minimization of the Debye–Waller factor. The Pd-loaded MOF and pure MOF samples weighed 500 mg and 2.5 g, respectively.

### Catalyst Testing

The catalyst (170 mg) with a sieve fraction of 250 – 425 μm was loaded inside a reactor tube (7 mm diameter), placed inside a tubular furnace, and connected with gas lines through Swagelok fittings. The samples were initially treated with H_2_ in order to reduce any possible PdO to Pd^0^ (30 min under H_2_ atmosphere at 323 K). After treatment, the catalysts were brought to room temperature (∼298 K), and the reaction was performed using a space velocity of 22500 h^–1^ with a reaction mixture composed of hydrogen (4% in helium, BOC Ltd.) and 1,3-butadiene (1% in helium, BOC Ltd.) in a percentage ratio of 95.2/4.8, using helium to balance. The flow rates (in mL/min) were 150 H_2_, 30 butadiene, and 225 He. In order to obtain a direct correlation between the catalytic activity results and the *in situ* XAFS measurements, the catalysts were regenerated in H_2_ (30 min at 323 K), and then the catalytic test was performed at 353 K. The activity and selectivity were monitored using online MS and GC. The GC was calibrated using a calibration mixture comprising (0.05% *cis*-2-butene, 0.15% *trans*-2-butene, 0.1% *n*-butane, 0.3% 1-butene, all balanced in He; BOC). For all the catalysts tested, the first GC measurement was performed 10–15 s after the online MS was able to detect the first reaction products. The GC sampling time was 0.3 h.

### X-ray Absorption Spectroscopy

The measurements were performed on the B18 beamline at the Diamond Light Source at the palladium K-edge (24.35 keV) and in transmission mode for 75.7 s per scan. The catalyst was loaded into a capillary and mounted on a catalyst test rig with the capillary connected to the gas lines on one side and a residual gas analyzer (in the MS) on the other side. Different gases, 10% H_2_ in He, 1% 1,3-butadiene in He, and a mixture of the two (2:98 in relative %), were passed over the catalysts, and XAFS spectra were recorded after the samples were kept under steady state for 20 min at each gas composition. Data processing and analysis were carried out using the Athena and Artemis software from the Demeter IFEFFIT package. The FEFF6 code was used to construct theoretical EXAFS signals, which included single-scattering contributions from atomic shells through the nearest neighbors in the face-centered cubic (FCC) structure of Pd. The *k*-range used for fitting was 3 to 12 Å^–1,^ and the *r*-range was from 1.15 to 3 Å. The path degeneracy was allowed to vary in the fit in order to account for the size effects that caused the surface atoms to be less coordinated than those in the particle interior. The amplitude reduction factor (Figure SI9) was fixed at 0.860, as obtained from the fitting of bulk Pd foil.

### Transmission Electron Microscopy

We used an aberration-corrected JEOL GrandARM 300F operated at a 300 kV accelerating voltage for imaging. A 20 μm probe-forming aperture was used, providing a probe convergence half-angle of 16.7 mrad and 4.8 pA beam current. Using a nominal camera length of 8 cm, we collected the signal from an annular dark-field (ADF) detector with scattering collection ranges of 80 to 240 mrad, forming a STEM image dominated by *Z* (average atomic number)-contrast.

**X-ray diffraction** (XRD) patterns were obtained using a Bruker D8 Discovery diffractometer with Cu *K*_α_ radiation (λ = 1.5418 Å). The measurements were conducted on a pressed powder sample holder, covering a 2θ range from 2° to 50°, with a step size of 0.02°/s under ambient conditions.

**Nitrogen adsorption** isotherms at −196 °C were recorded using a BELSORP mini-II instrument, and the specific surface area of the sample was calculated using the BET (Brunauer–Emmett–Teller) theory. The samples were pretreated under dynamic vacuum conditions at 80 °C for 1 h and then at 150 °C for 2 h prior to analysis.

**Microwave plasma emission spectroscopy** (MP-AES) was employed to determine the elemental composition of the samples using an Agilent 4100 instrument measuring zirconium at a wavelength of 339.198 nm and palladium at 360.955 nm.

## Computational Details

### Atomistic Modeling

All simulations were performed at the Density Functional Theory level with plane waves using Quantum Espresso version 6.8.^53,55–58^ Spin polarization was employed, with a kinetic energy cutoff of 50 Ry and 480 Ry for density and ultrasoft potentials, respectively. We used the pseudopotentials H.pbe-rrkjus_psl.1.0.0.UPF, N.pbe-n-rrkjus_psl.1.0.0.UPF, C.pbe-n-rrkjus_psl.1.0.0.UPF, O.pbe-n-rrkjus_psl.1.0.0.UPF, Zr.pbe-spn-rrkjus_psl.1.0.0.UPF, and Pd.pbe-spn-rrkjus_psl.1.0.0.UPF from http://www.quantum-espresso.org. All calculations were performed at the γ point. The clusters were embedded into a cubic box of 20 Å lattice with periodic boundary conditions. Phonon calculations were performed using phonopy^59,60^ starting from well-optimized geometries. The energy convergence criterion for ionic minimization was 1.0 × 10^–6^ Ry, and that for force was 1.0 × 10^–5^ Ry/Bohr. Phonon spectra corrections for comparison with INS were performed using the AbIns postprocessing code.^61^ Binding energies were computed by computing the total energy of the cluster plus hydrogen, from which we subtracted the total energy of the cluster and the energy of individual hydrogen atoms. Inputs and outputs were processed using Atomistic Simulation Environment python framework scripts.^62^

### Cluster Case

Our model predicts the maximum hydrogen load as 24 H atoms per Pd_17_ cluster with an almost constant binding energy per H atom. This coverage is significantly higher than the 2 H to 3 Pd ratio in the interstitial β-phase. We note, however, that even in the case of ‘overloading’, hydrogen was still found to populate the available hollow positions, i.e., all the 24 surface sites, rather than penetrating into the subsurface. The binding energy per H atom added to the surface is almost constant (ranging between 0.35 and 0.5 eV) up to 24 H atoms. The details of the binding energy are shown in Figure SI5. Furthermore, we analyzed the Pd–Pd average first-shell distance in the cluster under H loading, and found that it increased from 2.74 Å in a pure cluster to 2.86 Å in the cluster loaded with 24 atoms.

## Supplementary Material







## Notes

Media 1 (MOV)
Media 2 (MP4)
Computed charge density difference of the empty and Pd-loaded MOF, TDS spectra at different gas loading conditions, computed available H-adsorption sites on Pd_17_, INS spectra of Pd nanoparticles in vacuo and under H_2_ pressure, calculated binding energies of H on Pd_17_, computed phonon spectra, nearest-neighbor and Pd–Pd distance simulations in Pd_17_, calibration for XAS, and tabulated EXAFS fits for the Pd-loaded MOF; the videos display the *ab initio* molecular dynamics of the Pd17 cluster filled with 2 H atoms, starting with H inside the cluster at 300 K; the simulation time was 2 ps, during which both H atoms migrated to the surface, indicating no significant energetic barrier (PDF)

## Abbreviations

NC: nanocluster
MOF: metal–organic framework
TEM: transmission electron microscopy
EXAFS: extended X-ray absorption fine structure analysis
CN: coordination number
DFT: density functional theory
INS: inelastic neutron scattering
XANES: X-ray absorption near-edge structure
GC: gas chromatography
MS: mass spectrometry
XAFS: X-ray absorption fins structure
DMF: dimethylformamide
FCC: face-centered cubic
