# Supplementary material for: Functionalized MOFs for Subnanometric Control of Pd Speciation for Selective Hydrogenation of Butadiene
Source: ACS Appl Nano Mater. 2026 Jun 15;9(25):11720–30. doi: 10.1021/acsanm.6c00899 (PMC13316868; doi:10.1021/acsanm.6c00899)
Supplement: Supplementary file 3 [file an6c00899_si_003.pdf]

Supporting Information

Functionalized MOFs for Sub-nanometric Control of Pd Speciation for the Selective Hydrogenation of Butadiene

Donato Decarolis[a], Sahra Ahmed[b], James King[c], Alin-Marin Elena[d], Linda Zhang[e], Jeff Armstrong[f], Ines Lezcano-Gonzalez[g,h], Mohsen Danaie[i], Michael Hirscher[e,j], Simone Meloni[k], Andrew M. Beale[g,h], Petra Á. Szilágyi[b]*

[a]Diamond Light Source, Didcot OX11 0DE, U.K

[b]Centre for Materials Science and Nanotechnology (SMN), Department of Chemistry, University of Oslo, P.O. Box 1033, Blindern, N-0315 Oslo, Norway

[c]Queen Mary University of London, School of Engineering and Materials Science, Mile End Road, E1 4NS London, UK4

[d]Scientific Computing Department, Science and Technology Facilities Council, Daresbury Laboratory, Keckwick Lane, Daresbury, WA4 4AD, United Kingdom

[e]Advanced Institute for Materials Research (WPI-AIMR), Tohoku University, Sendai 980-8577, Japan

[f]ISIS Pulsed Neutron and Muon Facility, Science and Technology Facilities Council, Rutherford Appleton Laboratory, Didcot, OX11 0QX, UK

[g]Department of Chemistry, University College London, 20 Gordon Street, London, WC1H 0AJ, UK

[h]Research Complex at Harwell (RCaH), Harwell, Didcot, Oxfordshire, OX11 0FA, UK

[i]Diamond Light Source Ltd. electron Physical Science Imaging Centre (ePSIC), Harwell Science & Innovation Campus, Didcot, Oxfordshire, OX11 0DE

[j]Max Planck Institute for Intelligent Systems Solid State Research, D-70569 Stuttgart, Germany.

[k]Department of Chemical, Pharmaceutical and Agricultural Sciences, University of Ferrara, via Luigi Borsari 46, 44121 Ferrara, Italy

p.a.szilagyi@kjemi.uio.no

Classification: Public

Table of Contents

Figure SI1: Density Functional Theory calculations between NH_2-UiO-66and Pd⊂NH_2-UiO-66 2

Figure SI2: Thermal-desorption spectra of a hydrogen-deuterium 1:1 mixture in Pd⊂NH_2-UiO-66......2

Figure SI3: Available positions for H in/on the Pd_{17} cluster 3

Figure SI4: Inelastic neutron scattering spectra for Pd nanoparticles..3

Figure SI5: Calculated binding energy for Pd_{17} cluster 4

Figure SI6: Computed phonon spectra for the NH_2-UiO-66 and Pd⊂NH_2-UiO-66.................................4

Figure SI7: Pd *K* edge XANES for Pd⊂NH_2-UiO-66 5

Table SI1: EXAFS Fit results for Pd⊂NH_2-UiO-66 5

Figure SI8: Nearest neighbor Pd-Pd distance in Pd⊂NH_2-UiO-66...6

Figure SI9: Computed average Pd-Pd distance in the free Pd_{17} cluster 6

Figure SI10: HAADF-STEM images of Pd⊂NH_2-UiO-66 7

Figure SI11: PXRD diffractograms of NH_2-UiO-66and Pd⊂NH_2-UiO-66 7

Figure SI12: Nitrogen adsorption isotherms of NH_2-UiO-66and Pd⊂NH_2-UiO-66.................................8

Table SI2: Total pore volumes of NH_2-UiO-66 and Pd⊂NH_2-UiO-66 8

Table SI3: MPAES analysis of Pd content in Pd⊂NH_2-UiO-66 8

Table SI4: MPAES analysis of Pd content in Pd⊂NH_2-UiO-66...8

Table SI5: MPAES analysis of Pd content in Pd⊂NH_2-UiO-66...9

Table S6: MPAES analysis of Pd content in Pd⊂NH_2-UiO-66...9

References...9

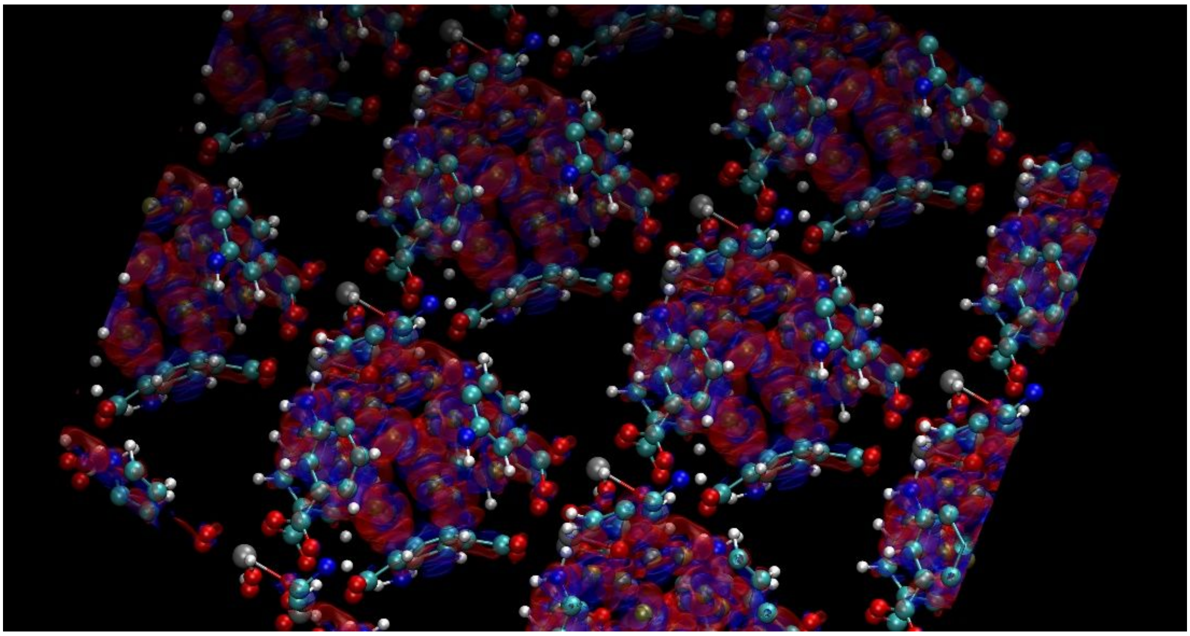

Figure SI1. Computed charge density difference from DFT calculations between the empty and Pd-laden MOF displaying pore loading. Blue indicates charge accumulation on the linkers being transferred from the Pd NC.

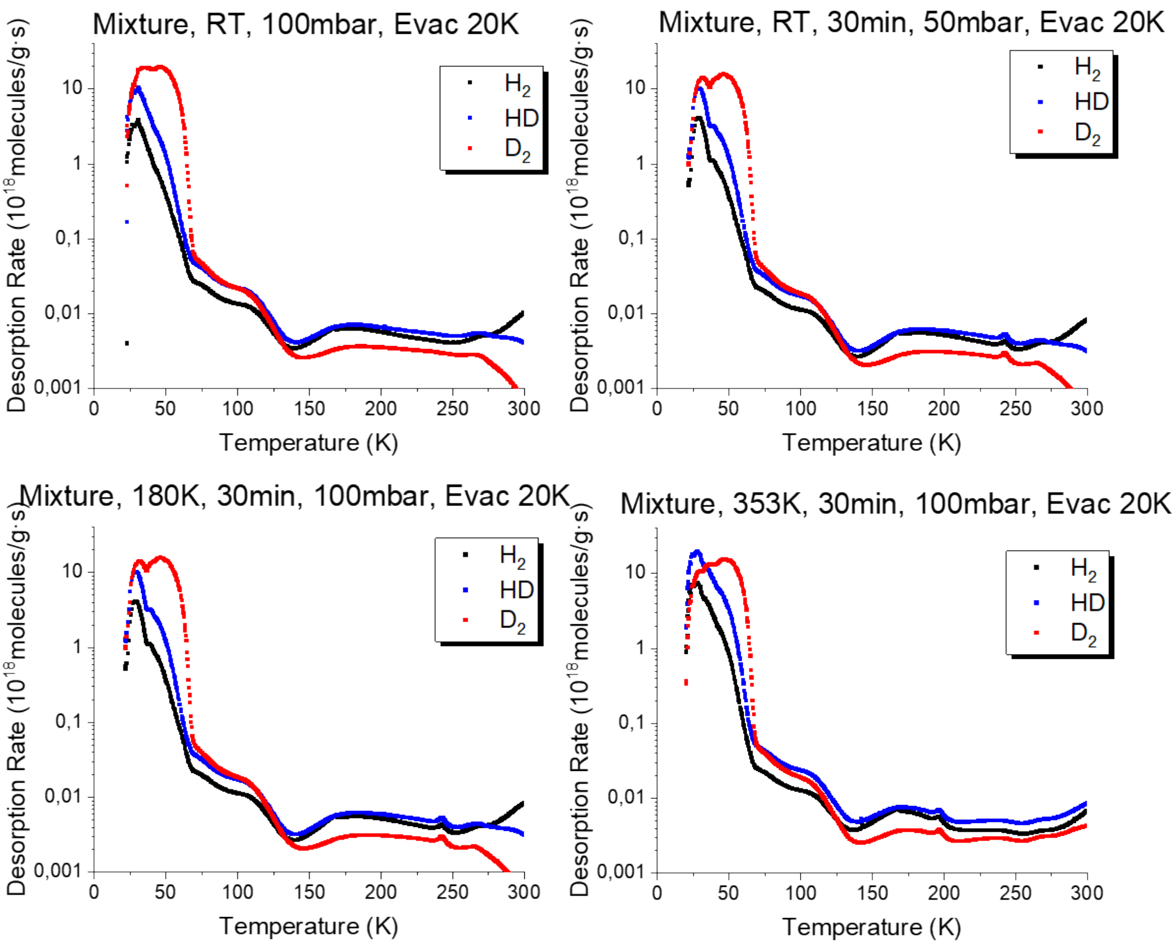

Figure SI2. Thermal-desorption spectra of a hydrogen-deuterium 1:1 mixture at different gas loading conditions.

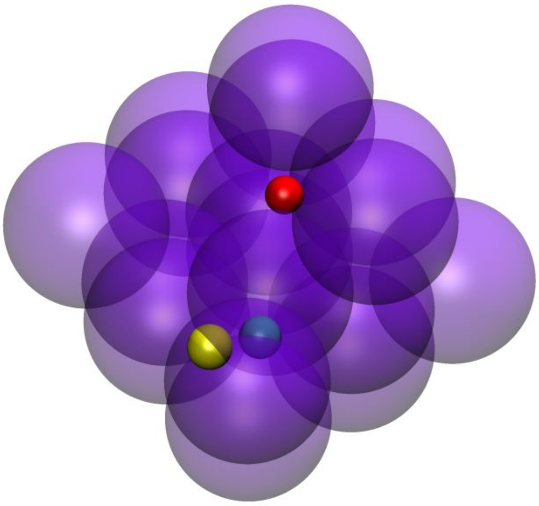

Figure SI3. Available positions for H in/on the Pd_{17} cluster, hollow positions in yellow, inside octahedra containing the central Pd, cyan and on top red. Binding energies are E_b=-0.35 eV for hollow position, E_b=-0.41eV for on top position and E_b=-0.06eV for inside position.

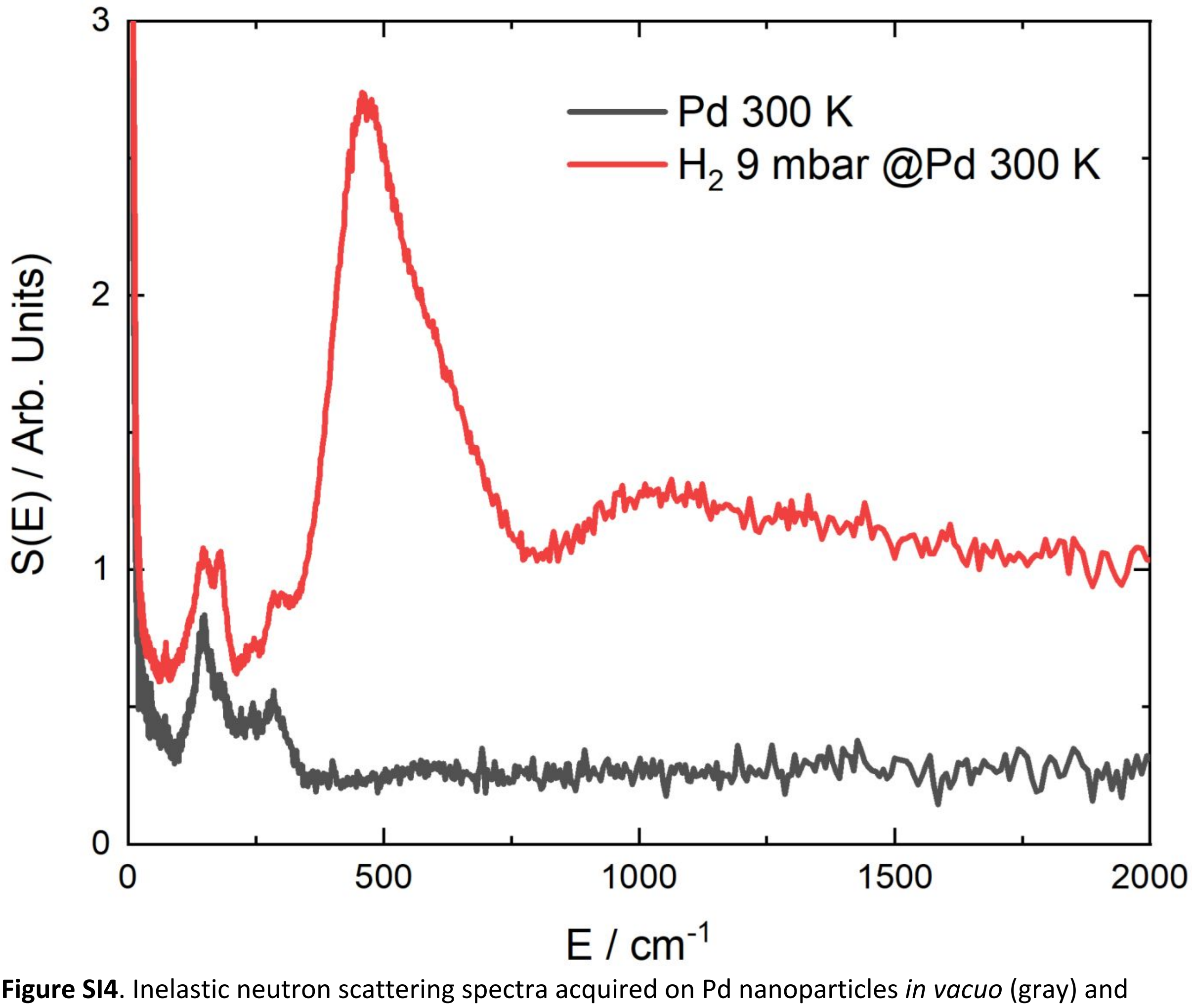

Figure SI4. Inelastic neutron scattering spectra acquired on Pd nanoparticles *in vacuo* (gray) and under 9 mbar hydrogen pressure (red) showing the characteristic peak for the interstitial hydride[1] at around 450 cm^{-1}.

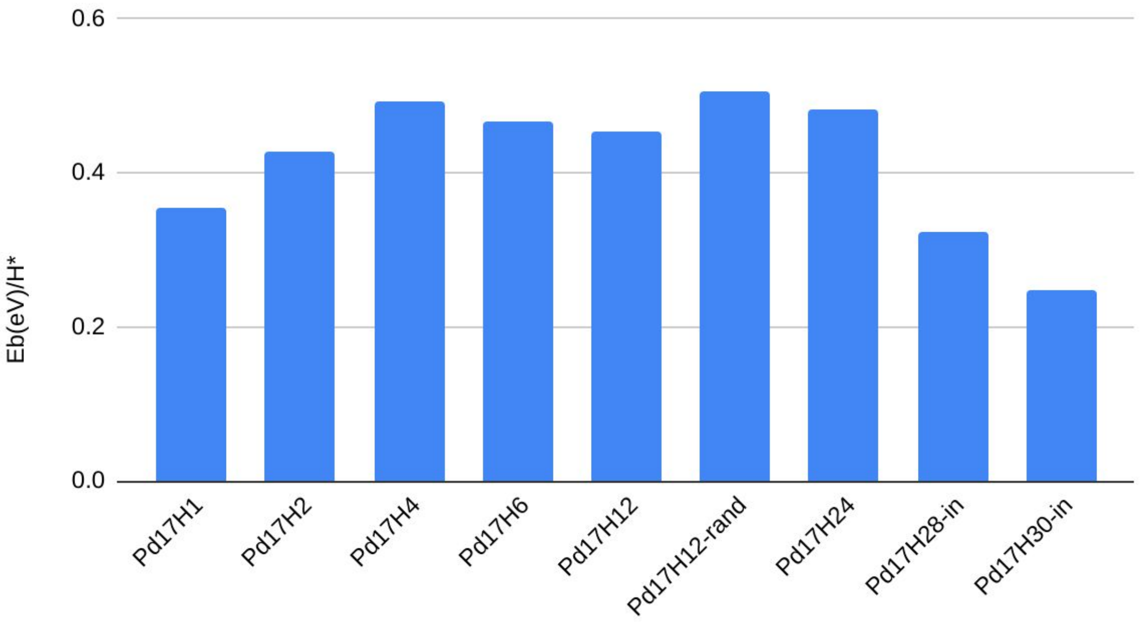

Figure SI5. Calculated binding energy for Pd_{17} cluster under H loading per Pd atom.

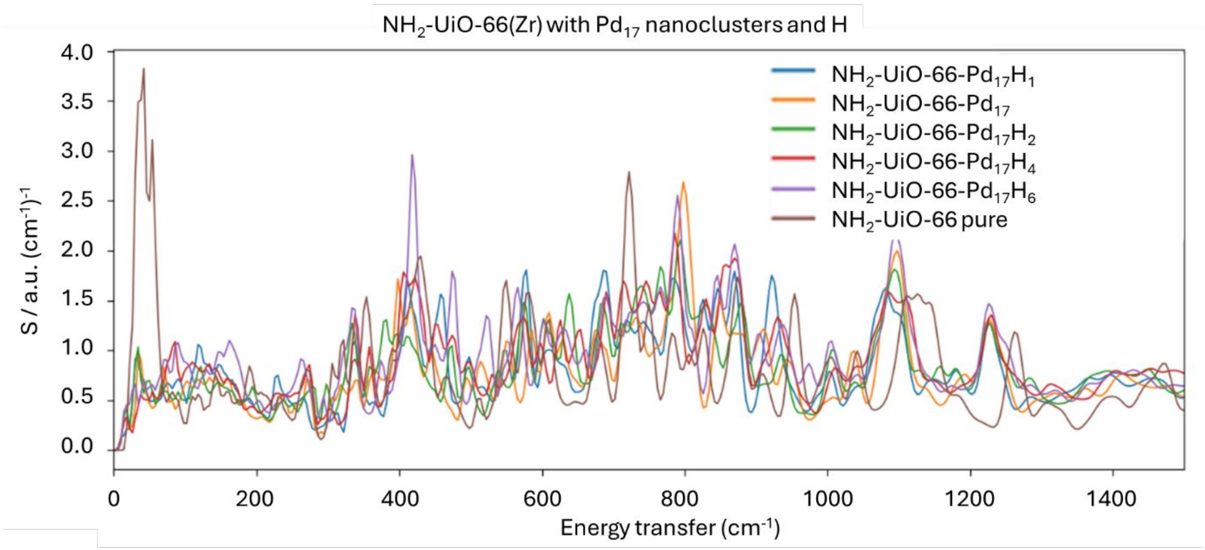

Figure SI6. Computed phonon spectra for the empty and the Pd_{17}-laden NH_2-UiO-66 at different hydrogen loadings.

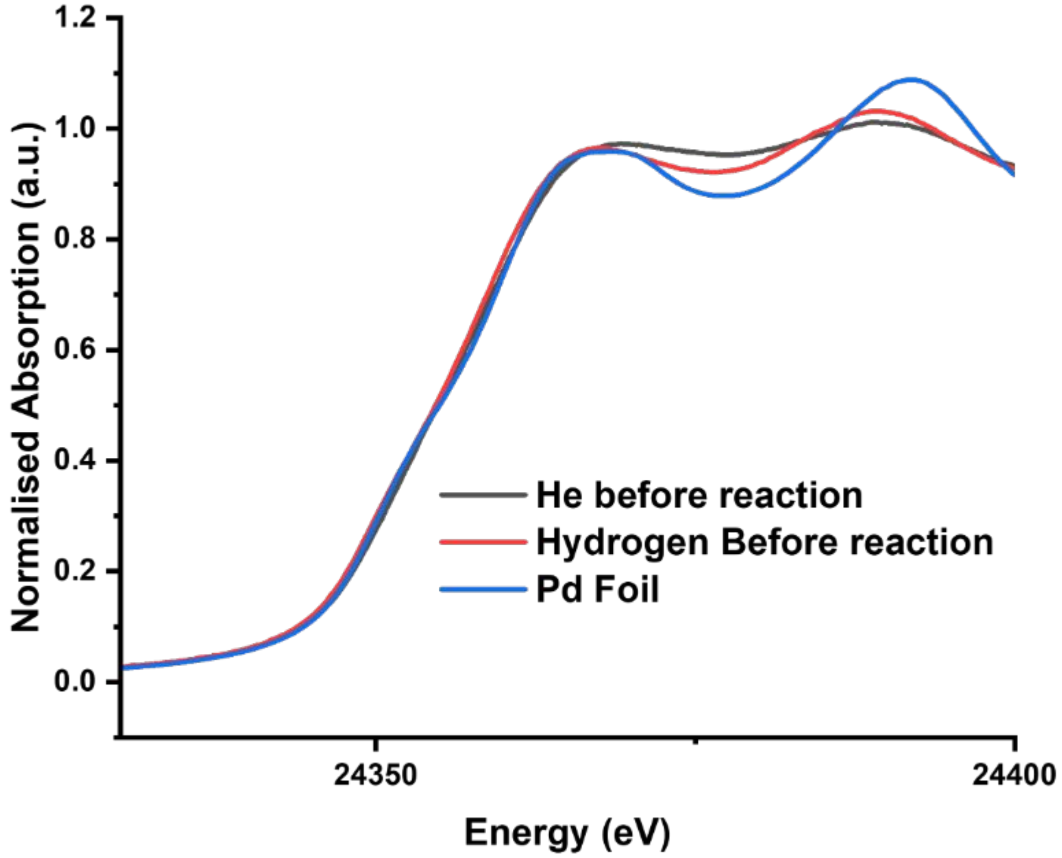

Figure SI7. Pd *K* edge XANES collected as prepared and after interaction with H_2, compared to a Pd foil.

Table SI1. EXAFS Fit results for Pd-laden NH_2-UiO-66 at different gas atmospheres.

**Gas Atmosphere**	**CN_{Pd-N} (a.u.)**	**σ^2_{Pd-N} (Å^2)**	**Radial $Distance_{Pd-N}$ (Å)**	**CN_{Pd-Pd} (a.u.)**	**σ^2_{Pd-Pd} (Å^2)**	**Radial $Distance_{Pd-Pd}$ (Å)**
He before reaction	2.05 ± 1	0.02 ± 1	2.121 ± 0.07	5.7 ± 0.7	0.009 ± 0.001	2.745 ± 0.007
Hydrogen Before reaction	-	-	-	8±1	0.00925 ± 0.0009	2.786 ± 0.006
Butadiene Before reaction	-	-	-	7.1 ± 0.9	0.009 ± 0.001	2.744 ± 0.007
Hydrogen/Butadiene	-	-	-	8 ± 1	0.0094 ± 0.0009	2.783 ± 0.006
He after reaction	-	-	-	7.4 ± 0.9	0.009 ± 0.001	2.745 ± 0.007
He After heating	-	-	-	8 ± 1.5	0.009 ± 0.001	2.77 ± 0.01

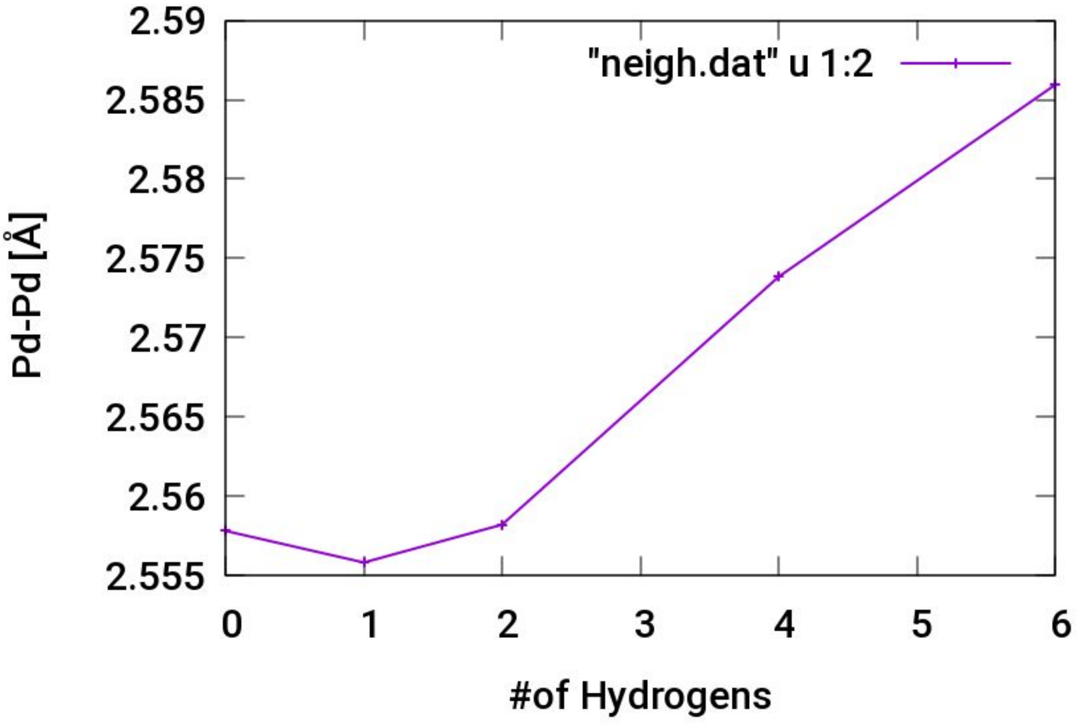

Figure SI8. Nearest neighbor Pd-Pd distance in a Pd_{17}NC embedded into the pores of NH_2-UiO-66 at different H loadings, computed from atomistic simulations.

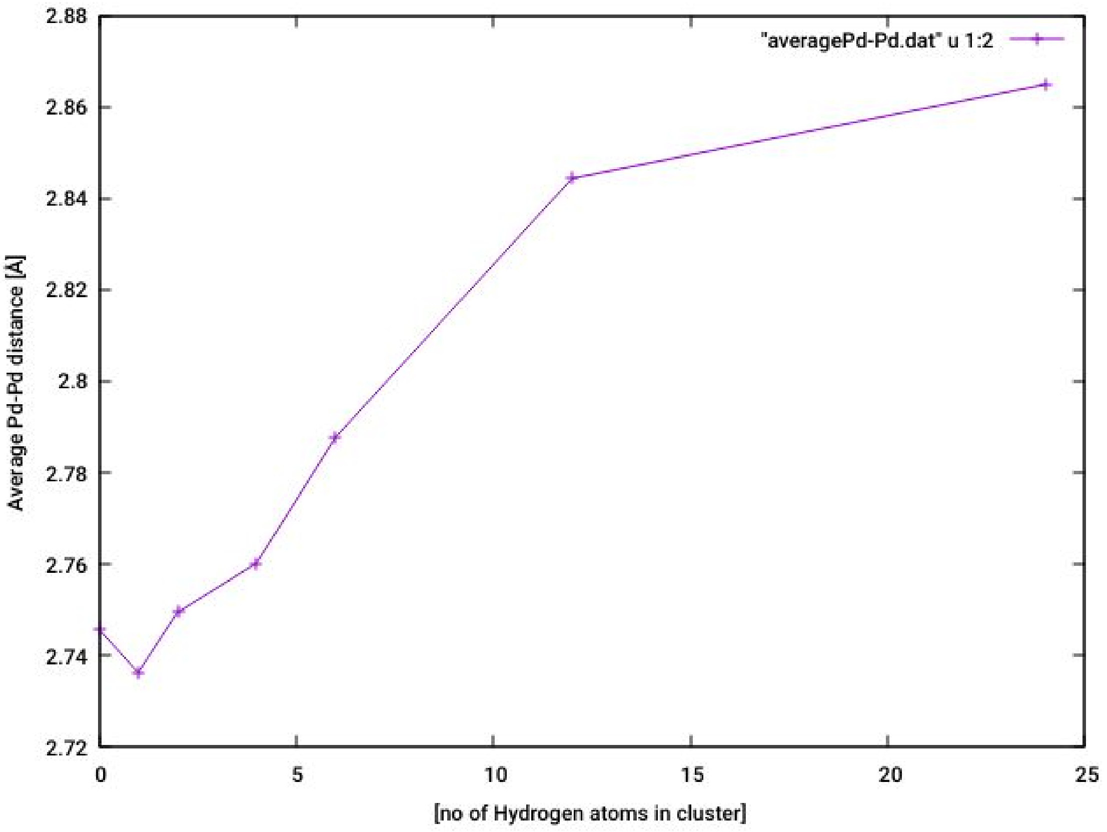

Figure SI9. Computed average Pd-Pd distance in the free Pd_{17} cluster at different H loadings.

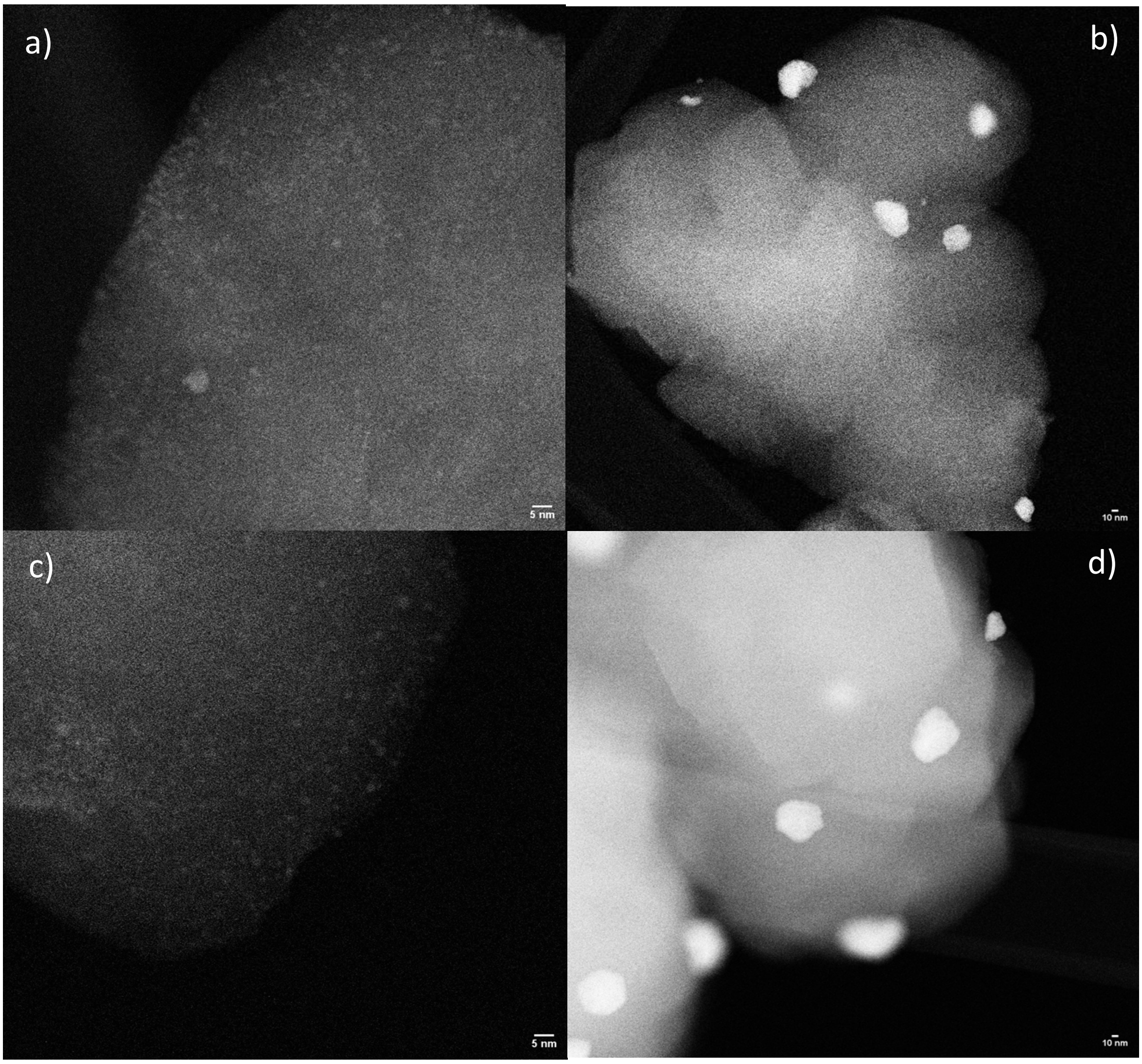

Figure SI10. HAADF-STEM images of Pd in NH_2-UiO-66 a,b) post-reduction and c,d) after aging, *i.e.* heat treatment at 240 °C in He, showing some degree of Pd nanoparticle formation on the MOF surface of up to 10 nm in b) and d), while the vast majority of Pd particles remain <1 nm, *i.e.* nanoconfined in the MOF pores.

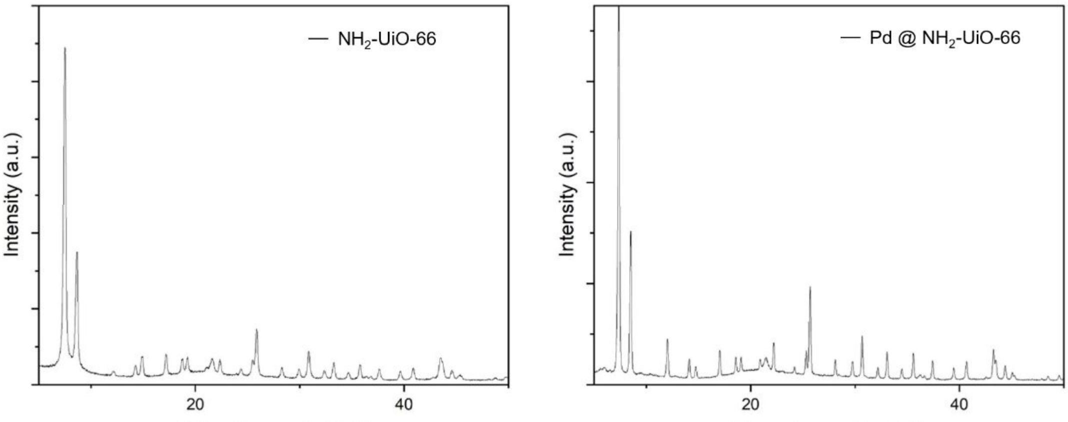

Figure SI11. PXRD diffractograms of NH_2-UiO-66 (left) and Pd in NH_2-UiO-66 (right).

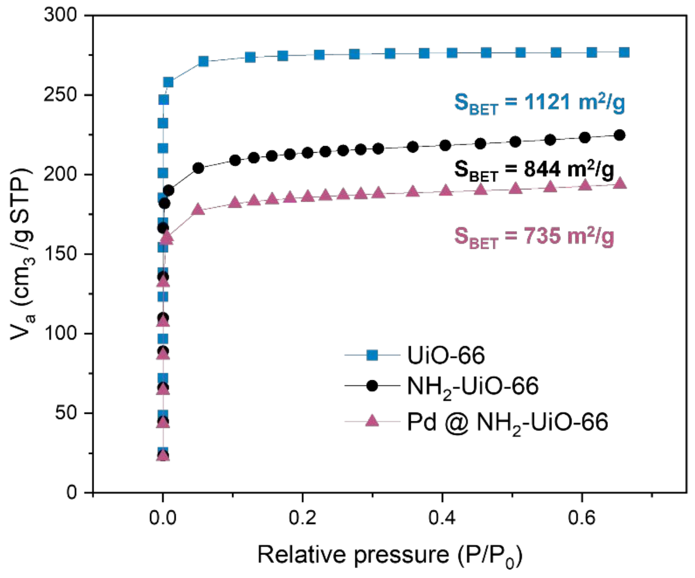

Figure SI12. Nitrogen adsorption isotherm (77 K) with specific surface areas of NH_2-UiO-66 and Pd⊂NH_2-UiO-66, with pristine UiO-66 shown for comparison.

Table SI2. Total pore volumes of NH_2-UiO-66 and Pd⊂NH_2-UiO-66 determined from N_2 adsorption isotherms (77 K), with pristine UiO-66 listed for comparison.

Sample	Pore volume (cm^3/g)
UiO-66	0.427
NH_2-UiO-66	0.365
Pd⊂NH_2-UiO-66	0.313

Table SI3. Concentrations of Zr (λ= 343.823 nm) and Pd (λ = 360.955 nm) in digested NH_2-UiO-66-Pd, obtained by microwave plasma atomic emission spectroscopy (MP-AES), and performed for a triplicate of samples with each sample measured three times.

Replicate	Zr [ppm]	Pd [ppm]	Pd/Zr [ppm]
1	67.2 ± 0.4	6.51 ± 0.06	0.498 ± 0.006
2	66 ± 1	7.67 ± 0.04	0.60 ± 0.01
3	69.6 ± 0.4	6.75 ± 0.07	0.498 ± 0.006
**Average**			**0.53 ± 0.01**

Table SI4. Concentrations of Zr (λ= 343.823 nm) and Pd (λ = 363.470 nm) in digested NH_2-UiO-66-Pd, obtained by MP-AES, and performed for a triplicate of samples with each sample measured three times.

Replicate	Zr [ppm]	Pd [ppm]	Pd/Zr [ppm]
1	67.2 ± 0.4	6.8 ± 0.1	0.52 ± 0.01
2	66 ± 1	7.79 ± 0.02	0.61 ± 0.01
3	69.6 ± 0.4	6.4 ± 0.2	0.48 ± 0.02
**Average**			**0.54 ± 0.01**

Table SI5. Concentrations of Zr (λ= 339.198 nm) and Pd (λ = 363.470 nm) in digested NH_2-UiO-66-Pd, obtained by MP-AES, and performed for a triplicate of samples with each sample measured three times.

Replicate	Zr [ppm]	Pd [ppm]	Pd/Zr [ppm]
1	80.0 ± 0.9	6.8 ± 0.1	0.44 ± 0.01
2	78 ± 1	7.79 ± 0.02	0.517 ± 0.009
3	90.0 ± 0.6	6.4 ± 0.2	0.48 ± 0.01
**Average**			**0.48 ± 0.01**

Table SI6. Concentrations of Zr (λ= 339.198 nm) and Pd (λ = 360.955 nm) in digested NH_2-UiO-66-Pd, obtained by MP-AES, and performed for a triplicate of samples with each sample measured three times.

Replicate	Zr [ppm]	Pd [ppm]	Pd/Zr [ppm]
1	80.0 ± 0.9	6.51 ± 0.06	0.418 ± 0.006
2	78 ± 1	7.67 ± 0.04	0.509 ± 0.009
3	90.0 ± 0.6	6.75 ± 0.07	0.428 ± 0.0005
**Average**			**0.451 ± 0.005**

References

(1) Antonov, V. E.; Fedotov, V. K.; Ivanov, A. S.; Kolesnikov, A. I.; Kuzovnikov, M. A.; Tkacz, M.; Yartys, V. A. Lattice Dynamics of High-Pressure Hydrides Studied by Inelastic Neutron Scattering. *J. Alloys Compd.* **2022**, *905*, 164208.
